# Comparison of Retrospective Motion Compensation Techniques for Pulmonary Dynamic Ultrashort Time to Echo MRI in Suspected Idiopathic Pulmonary Fibrosis

**DOI:** 10.1002/jmri.70350

**Published:** 2026-05-23

**Authors:** Abhilash S. Kizhakke Puliyakote, Luis A. Torres, Aiah AlAtoum, Natally AlArab, Kevin M. Johnson, Rodrigo M. Bello, Bryan O'Sullivan‐Murphy, Joseph G. Mammarappallil, Andrew D. Hahn, Sean B. Fain

**Affiliations:** ^1^ Department of Radiology University of Iowa HealthCare Iowa City Iowa USA; ^2^ Department of Medical Physics University of Wisconsin‐Madison Madison Wisconsin USA; ^3^ Roy J. Carver Department of Biomedical Engineering University of Iowa Iowa City Iowa USA; ^4^ Department of Radiology Duke University School of Medicine Durham North Carolina USA

**Keywords:** free‐breathing, IPF, pulmonary MRI, respiratory motion compensation, retrospective gating, UTE‐MRI

## Abstract

**Background:**

Motion can degrade image quality during Ultrashort Time‐to‐Echo (UTE) pulmonary MRI and is particularly prevalent in patients with lung disease. Comprehensive assessment of the impact of motion compensation techniques on image quality and clinical interpretation is needed.

**Purpose/Hypothesis:**

To compare the impact of retrospective motion compensation schemes on image quality and clinical interpretation of pulmonary UTE MRI in idiopathic pulmonary fibrosis (IPF).

**Study Type:**

Prospective.

**Population:**

21 (male = 18; mean age, 69.9 ± 8.1 years) participants with suspected IPF.

**Field Strength/Sequence:**

1.5 T/3 T, 3D center‐out radial (gradient‐echo) UTE sequence with 2× radial oversampling, while free‐breathing.

**Assessment:**

Images were reconstructed to 1.25 mm isotropic resolution using five retrospective schemes: no gating, hard‐gating, soft‐gating, motion‐resolved (XD‐GRASP), and an iterative approach (iMoCo). Signal‐to‐noise ratios (SNR) were estimated within the lung parenchyma, airways, aorta, muscles, and liver. Contrast‐to‐noise ratios (CNR) were estimated using the mean airway signal as reference. Image sharpness was estimated using the maximum derivative of a line profile across the diaphragm and a wavelet‐based autofocus measure. Three radiologists evaluated image quality, motion artifacts on a 5‐point Likert scale, and diagnostic classification of usual interstitial pneumonia (UIP).

**Statistical Tests:**

The Kruskal–Wallis non‐parametric test was used for qualitative reader scores and one‐way ANOVA for the quantitative metrics, with *p* < 0.05 as the threshold for significance.

**Results:**

CNR was highest using the iMoCo reconstructions (lung parenchyma: 1.64 ± 1.41 vs. 0.88 ± 0.81 via XD‐GRASP). Image sharpness was significantly improved using compressed sensing (CS)‐based techniques (XD‐GRASP and iMoCo), compared to the other methods, using both diaphragm profile (CS: 6.28 ± 3.70 vs. non‐CS: 3.73 ± 2.06) and wavelet metrics (CS: 2.33 ± 0.42 vs. non‐CS: 2.05 ± 0.35). CS methods also demonstrated greatest image quality based on reader scores.

**Conclusion:**

Motion compensation using compressed sensing methods can improve image quality and clinical utility of UTE‐MRI in the identification and diagnostic classification of typical parenchymal fibrotic patterns.

**Evidence Level:**

Level 2—Prospective study, with a reference standard determined during the course of the study (CT imaging).

**Technical Efficacy:**

Stage 1.

## Plain Language Summary

Patients with fibrotic lung disease have difficulty holding their breath for scans. Some MRI motion correction techniques allow patients to breathe freely during imaging, recovering image quality after the scan. We compare five techniques to minimize the impact of motion, using two approaches: first using a set of measurements calculated mathematically from the images, and second by having a trio of Radiologists score and interpret them clinically. We show that the advanced algorithms can improve the quality of images and allow doctors to evaluate their patients without the need for radiation, making pulmonary MRI application practical for clinical use.

## 1 Introduction

Idiopathic Pulmonary Fibrosis (IPF) is a chronic, irreversible, progressive interstitial lung disease (ILD) that affects approximately 3 million people worldwide [1, 2], with a prevalence rate of 96.2 per 100,000 in 2022 in the United States [3]. Diagnostic decision‐making relies on multidisciplinary evidence, using a combination of lung function testing, serum markers, and high‐resolution computed tomography (HRCT), along with patient demographics and medical history, using surgical biopsies as needed [4]. HRCT remains the most accurate non‐invasive test for IPF diagnosis, with a histological pattern of usual interstitial pneumonia (UIP), and subpleural and basal predominance of features including honeycombing, traction bronchiectasis, ground‐glass infiltrates, and reticulation [4]. While HRCT has clinical potential in characterizing the pattern and extent of fibrotic disease, these patterns have demonstrated limited application in differentiating stable and progressive disease or predicting response to therapy [5, 6]. The risks associated with cumulative radiation exposure further limit the utility of CT imaging for longitudinal monitoring and evaluating impact of therapy.

Pulmonary MRI applications have often been limited due to signal‐to‐noise (SNR) considerations. At functional residual capacity (FRC), only ~25% of the lung field is composed of tissue, yielding low net magnetization, especially in the non‐dependent lung with only ~10% tissue [7]. The changes in magnetic susceptibility at the air‐tissue interfaces in the lung parenchyma lead to high local field inhomogeneities [8, 9], resulting in very short transverse decay times (T_2_*) of approximately 2 ms and 0.5 ms at 1.5 T and 3 T, respectively [10], resulting in overall loss in local contrast, further impeding visualization of the above mentioned features such as honeycombing and reticulation. Ultrashort time‐to‐echo (UTE) MRI mitigates the rapid signal loss from T_2_* decay by sampling the free induction decay within an echo time (TE) of 50–200 μs after the radio frequency (RF) excitation [11]. Robust 3D UTE‐MRI has previously demonstrated clinical utility comparable to HRCT in various diseases in adults and neonates with the added benefit of not using ionizing radiation [12, 13, 14, 15, 16]. UTE‐MRI has demonstrated clear advantages to conventional approaches through SNR improvements in animal models [17], improved visualization of lung and airways in healthy controls and patients with fibrosis [18], T_1_/T_2_* mapping [9, 19], and in the diagnosis and monitoring of cystic fibrosis [20, 21, 22].

The low tissue density of the normal lung parenchyma makes signal averaging necessary to improve SNR for high resolution imaging of normal lung structures, even with UTE‐MRI. In the case of restrictive lung disease such as fibrosis, reduced ventilation and increased lung density may potentially increase the number of protons available for image formation. Image acquisition during free‐breathing allows for an increased number of projections, improving SNR through additional signal averaging [9, 23, 24], especially in patient cohorts with chronic lung diseases where breath‐holds are not feasible. However, longer acquisition times during respiratory motion require prospective motion triggering or retrospective gating. Image reconstruction in free‐breathing UTE‐MRI can be improved using gating to correct artifacts associated with respiratory motion [25, 26]. These free breathing acquisitions commonly use pseudo‐random view ordering, such as Golden Angle [27] or “bit reversal” [28], to mitigate image artifacts due to motion. The projection data can then be “binned” into temporal or respiratory/cardiac motion states, while maintaining pseudo‐uniform *k*‐space coverage. Image reconstruction may utilize a partial or full set of projections, using different respiratory phase windows [29, 30], or use image registration and compressed sensing to iteratively reconstruct images with reduced motion artifacts and improved image resolution [31, 32, 33].

Previous work has demonstrated clear evidence of improved sharpness of pulmonary MRI using motion compensation in 3D UTE‐MRI applications in neonatal and pediatric applications [25]. However, there remains the need to evaluate the performance of these motion correction schemes in adult patients with pulmonary disease, and to further investigate the relative impact of these techniques on the evaluation of structural parenchymal features by Radiologists. In this work, we aimed to investigate a range of respiratory motion mitigation methods in a patient population with IPF, undergoing 3D radial UTE MRI and their impact on image quality and the clinical utility for diagnosis and monitoring, with the goal of identifying the best approach for visualizing lung parenchymal texture features critical for diagnosis of IPF using UTE‐MRI.

## 2 Material and Methods

### 2.1 Study Population

The study was approved by the institutional review board (IRB: 2013‐0266 and 2014‐1572) at the UWHealth University Hospital, Madison, WI. Patients over the age of 18, with no recent history of exacerbations were prospectively recruited based on a likely diagnosis of IPF from the multi‐disciplinary ILD clinic in our hospital system for this study and provided written informed consent. Patients contraindicated for MRI, pregnant or lactating, or with other medical conditions that could interfere with the ability to comprehend and comply with the study directions were excluded. Some of the results in this work have been previously published as part of a doctoral dissertation [34].

### 2.2 Data Acquisition

Participants were scanned on a 1.5 T Signa HDx scanner (GE Healthcare, Waukesha, WI) at the start of the study. Following hardware upgrades, image acquisition continued on a 3 T Discovery MR750 system (GE Healthcare, Waukesha, WI). A total of 12 participants were scanned on the 1.5 T Signa, and nine on the 3 T Discovery.

UTE‐MRI imaging was performed with the patient in the supine position, during normal tidal breathing, using an 3D center‐out radial UTE sequence optimized for retrospective dynamic pulmonary reconstruction [18] (TE = 80–90 μs; TR = 2800–3400 μs, 90,000 spokes) with 2× radial oversampling to ensure full chest coverage with a target FOV of 40 cm, reconstructed to isotropic resolution of 1.25 mm over a 32 cm FOV.

### 2.3 Image Reconstruction

The signal at the center of *k*‐space (DC component) was obtained from the coil element with the highest absolute deviation from the mean DC signal and served as a surrogate for respiratory motion. The respiratory motion navigator was extracted as the output of a bandpass filter (~1 Hz bandwidth) on this signal [24]. For images acquired at 3 T, coil compression using the principal components approach was applied to match the 1.5 T acquisition with 8 coils [35]. Re‐gridding with density compensation was used to correct for the non‐uniform *k*‐space sampling with the radial trajectory [36], followed by reconstruction to a 1.25 mm isotropic resolution using the following schemes:

1. *No Gating*: All *k*‐space data was included without considering the respiratory navigator.
2. *Hard Gating*: A step function was applied using the respiratory navigator to restrict reconstruction using *k*‐space data from the lower 50% of the respiratory cycle, using the end‐expiratory motion state as the reference.
3. *Soft Gating*: An exponential weighting function was used to prioritize *k*‐space data closer to the end‐expiratory reference. Data within 20% of the reference frame had a weight of 1, with exponentially decaying weights (decay factor of 0.8) for other frames [29, 30].
4. *Motion Resolved XD‐Grasp*: The respiratory navigator was used to bin the *k*‐space data into six motion states from end‐expiration to end‐inspiration, with compressed sensing using the eXtra‐Dimensional GRASP method. An iterative reconstruction was used to solve the XD‐GRASP cost function [31], with a fixed regularization penalty of 0.05.
5. *Iterative Motion Compensation (iMoCo)*: In the iMoCo approach, each motion frame from the XD‐GRASP approach was registered to the reference end‐expiratory frame. The estimated motion fields were used as prior information to solve for the iMoCo cost function, with a fixed spatial variation penalty parameter set to 0.05 across all datasets [32].

The last two techniques (XD‐GRASP and iMoCo) are collectively refered to as compressed sensing (CS)‐based techniques here. Selection of parameters used for the retrospective motion compensation techniques were based on previous experience in data reconstruction [16, 25, 34], and the source code is available on github at the following location: http://www.github.com/ltorres6/imoco. Bias field correction was applied using the N4 bias field correction algorithm [37].

### 2.4 Data Analysis

Image quality of each approach was evaluated quantitatively and qualitatively. For the quantitative analysis, regions of interest were manually drawn across regions in the liver, lung parenchyma, aorta, muscle, and airways. The SNR was estimated in each tissue using an equivalent volume region in the background. The contrast‐to‐noise ratio (CNR) was assessed relative to the signal in the airways as a low‐intensity reference. Image sharpness was evaluated using two different measures: across the lung/diaphragm interface by computing the maximum derivative within a manually drawn region of interest (by coauthor: LAT, a graduate student at that time) and normalizing to the liver signal; and a wavelet based autofocus measure [38, 39]. Given the diaphragmatic sharpness measure may be obfuscated by severe fibrotic patterns in the basal and posterior portions of the lungs typical in IPF, a wavelet‐based autofocus method was also used to evaluate sharpness. The wavelet‐based approach is less sensitive to local fibrosis patterns in IPF, quantifies the overall sharpness of the image, and eliminates potential user bias in selecting regions of interest.

To assess image representation of clinical patterns of disease, three radiologists (BOSM—7 years of experience, JGM—12 years of experience, RMB—21 years of experience) were blinded to the randomized datasets and scored each image on a 5‐point Likert scale, on metrics related to image quality, clinical relevance (diagnostic classification, degree of structural parenchymal abnormalities, confidence in scoring), and artifact severity (cardiac motion, respiratory motion, and other artifacts). The HRCT images were scored to rank the diagnostic classification of the IPF pattern as definite UIP (usual interstitial pneumonia), probable UIP, or NSIP (nonspecific interstitial pneumonia). A summary of the severity distribution and diagnostic classifications is presented in Table 1.

**TABLE 1:** Participant characteristics.

Count	21
3.0 T *N* (%)	9 (42.9)
Female *N* (%)	3 (14.3)
Age (years) Median (IQR)	72 (68.0, 75.5)
CT pattern (%)	Definite UIP (47.6%)
	Possible UIP (42.8%)
	NSIP (9.5%)
Predominant peripheral disease *N* (%)	20 (95.2)
Percent fibrotic lung Mean (SD)	22.4 (17.8)

Abbreviations: IQR, interquartile range; NSIP, non‐specific interstitial pneumonia; SD, standard deviation; UIP, usual interstitial pneumonia.

### 2.5 Reference Standard

Volumetric HRCT scans were acquired during the same visit, and provided a reference for structural comparisons of type and pattern of pulmonary fibrosis. The HRCT images were scored as a standard of reference for the MRI‐based diagnostic classification of the IPF pattern as definite UIP (usual interstitial pneumonia), probable UIP, or NSIP (nonspecific interstitial pneumonia). All HRCT images were anonymized (independently from UTE MRI images) and visually assigned a diagnostic pattern by a radiologist (RBM) in randomized order.

### 2.6 Statistical Analysis

A Kruskal–Wallis non‐parametric test was used to test for significant differences among the techniques. Quantitative metrics were analyzed using one‐way ANOVA tests. Statistical significance was tested using an alpha threshold of 0.05. Agreement between readers for each qualitative metric was assessed using Kendall's rank correlation coefficient (Kendall's tau *τ*). Qualitative scores were interpreted using the median score for each technique. Effect of field strength was tested using a linear mixed effects model with field strength as a covariate. Diagnostic classification scores from MRI were condensed from the 5‐point Likert Scale to a 3‐point scale (“unknown” and “not IPF” grouped as “NSIP,” “possible IPF” and “probable IPF” as “probable UIP,” and “definite IPF” as “definite UIP”) to match the CT‐based classification and compared using Spearman's rank correlation coefficient.

## 3 Results

A total of 21 participants (male = 18; mean age, 69.9 ± 8.1 years) completed the study successfully and represented a range of parenchymal fibrotic burden and severities. On average, 95.87% ± 2.57% of *k*‐space data was used for reconstruction without any motion correction and 47.94% ± 1.28% with hard‐gating. Further binning resulted in 24.21% ± 2.55% in the end‐expiratory bin for XD‐GRASP, and 93.33% ± 2.81% data used across all bins for both XD‐GRASP and the iMoCo reconstructions. The CS‐based methods are more computationally intensive, requiring ~24 h of run time on a high‐performance computing system with a dedicated GPU, compared to ~30 min runtime for the gridding‐based reconstructions.

**FIGURE 1:**
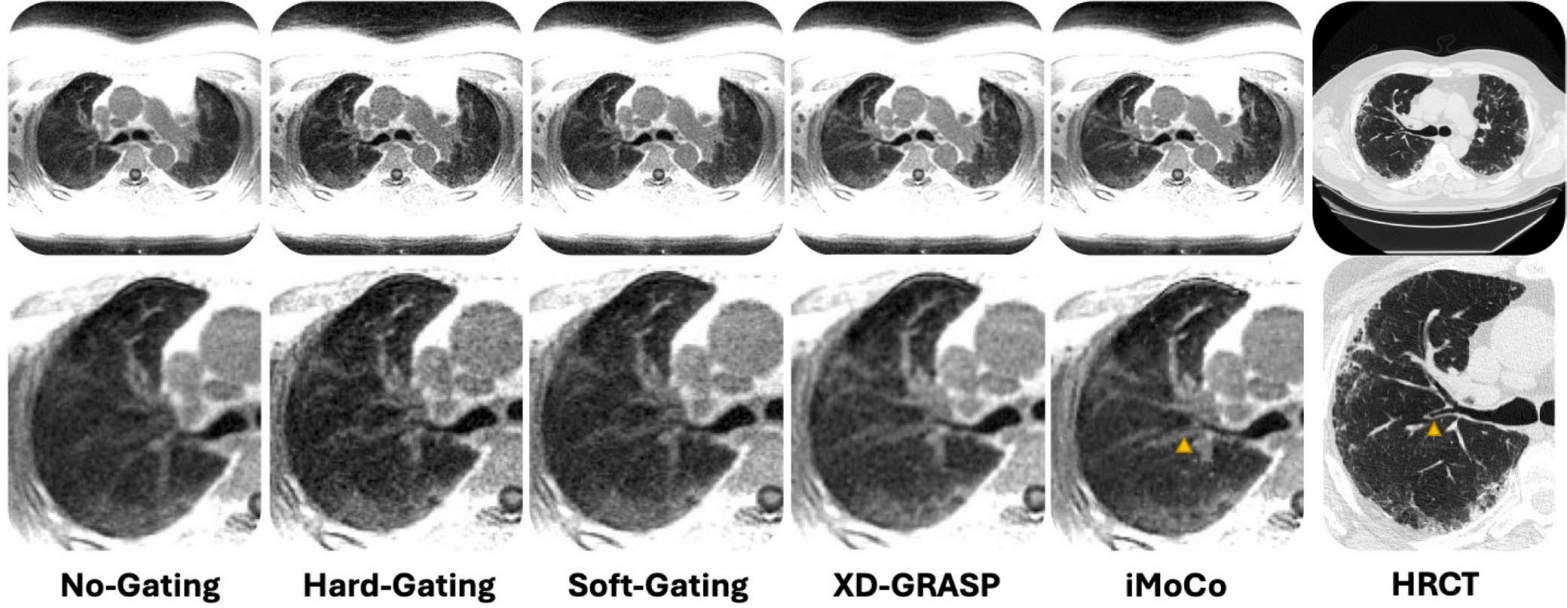
Representative slice from a patient diagnosed with IPF, with “possible UIP” pattern on CT, demonstrating visually evident improvement with advanced motion compensation schemes. An anatomically matched slice from high‐resolution CT is shown at the right for structural reference. The top row shows the complete slice, while the bottom row shows a closer view of the right lung to highlight differences. The orange arrow indicates location of an airway segment visible on CT, that can be identified using iMoCo reconstructions, but is obscured in other schemes. Fibrotic changes in the posterior lung are evident across all schemes to varying extents.

**FIGURE 2:**
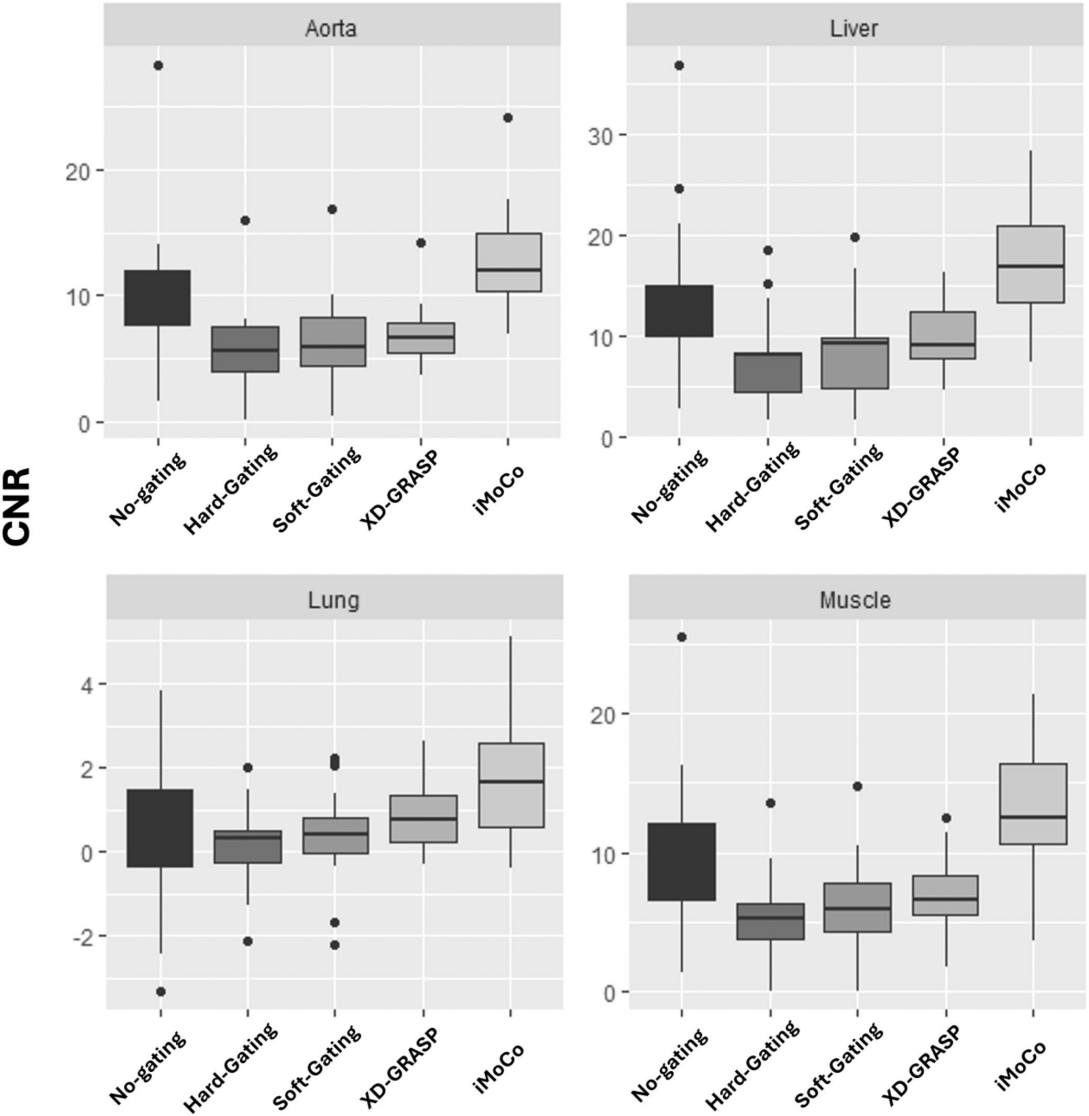
Distribution of contrast‐to‐noise ratio (CNR) across tissue types and reconstruction schemes. Contrast was defined using mean signal in a manually defined region with the airways as a low‐intensity reference and noise in the background. CNR was significantly improved using iMoCo reconstruction compared to all other schemes.

Sample images demonstrating image quality and textural differences between the five reconstruction schemes are shown in Figure 1. Plots of the distributions of the quantitative CNR metrics across all motion compensation schemes are shown in Figure 2. Across all tissues, CNR was significantly improved using the iMoCo approach (lung parenchyma: 10.74 with iMoCo vs. 5.86 with XD‐GRASP, and average of 5.70 across non‐CS methods). In general, contrast was reduced for hard‐gating, soft‐gating, and XD‐GRASP methods compared to the no‐gating approach, however improvements were demonstrated in iMoCo in all tissues as seen in Figure 2. The maximum derivative sharpness metric evaluated across the lung/diaphragm interface was significantly improved in XD‐GRASP and iMoCo relative to other methods (CS methods: 6.28 ± 3.70 × 10^−5^ vs. 3.73 ± 2.06 × 10^−5^ in non‐CS methods). The wavelet autofocus measure of sharpness measure showed a similar trend and indicated that XD‐GRASP and iMoCo generate the sharpest images (CS methods: 2.33 ± 0.42 × 10^−3^ vs. 2.05 ± 0.03 × 10^−3^ in non‐CS methods). A summary of the quantitative CNR and sharpness metrics is provided in Table 2.

**TABLE 2:** Summary of quantitative metrics of image quality.

	No‐gating	Hard‐gating	Soft‐gating	XD‐GRASP	iMoCo
CNR mean (SD)					
Aorta	9.92 (5.55)	5.60 (3.30)	6.42 (3.54)	7.04 (2.23)	12.82 (3.97)*
Liver	13.52 (7.44)	9.98 (4.31)	9.02 (4.62)	9.70 (3.21)	17.58 (5.96)*
Lung	0.66 (1.67)	0.31 (0.96)	0.41 (1.08)	0.88 (0.81)	1.64 (1.41)*
muscle	9.79 (5.37)	5.32 (2.92)	6.13 (3.25)	7.02 (2.64)	12.87 (4.53)*
Sharpness mean (SD)					
Maximum derivative (×10^−5^)	2.80 (1.43)	4.18 (2.15)	4.21 (2.25)	6.67 (3.89)**	5.89 (3.56)**
Wavelet Autofocus (×10^−3^)	2.01 (0.36)	2.07 (0.35)	2.07 (0.35)	2.34 (0.42)**	2.33 (0.43)**

*Note:* Bold values denote significance at *p* < 0.05. The * denotes variables where only iMoCo was significantly greater, whereas ** denotes variables with improvements observed using both CS methods.

Abbreviations: CNR, contrast to noise ratio; iMoCo, iterative Motion Compensation technique; SD, standard deviation; XD‐GRASP, eXtra dimensional golden‐angle radial sparse parallel technique.

**FIGURE 3:**
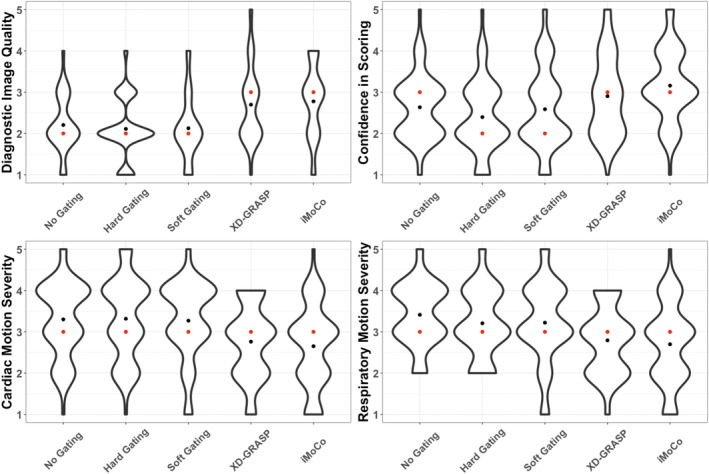
Density curves of reader score distributions. The distribution of overall image quality, confidence in diagnostic scoring, severity of cardiac motion, and severity of respiratory motion in each reconstruction scheme are shown in violin plots. Higher scores are better for image quality and confidence scores, while lower scores are better for motion severity. Compressed sensing (CS)‐based approaches have better (lower) motion severity scores suggesting improved motion compensation. Differences in overall image quality distributions are less evident in the distributions, but CS techniques outperformed the gridding‐based techniques in over 74% of cases.

There was significant agreement between readers on the rankings of motion compensation schemes across all metrics as shown in Table 3 (across motion mitigation schemes: Image quality: *τ* = 0.79 ± 0.09; Diagnostic classification: *τ* = 0.81; degree of pathology: *τ* = 0.82). A chart of the distribution of the reader scores for overall image quality, confidence in diagnostic classification, cardiac motion severity, and respiratory motion severity is shown in Figure 3. The mean and median scores for each motion compensation scheme are shown in black and red circles respectively. More directly relevant to clinical application, the CT‐based severity classification was significantly associated with the clinical classification derived from datasets reconstructed with iMoCo (Spearman's *R* = 0.54), XD‐GRASP (*R* = 0.63), and to a lesser degree without any gating (*R* = 0.37). Both soft‐gating (*R* = 0.50, *p* = 0.069) and hard‐gating (*R* = 0.33, *p* = 0.11) showed a trend but did not reach statistical significance.

**TABLE 3:** Inter‐reader agreement (quantified using Kendall's rank correlation coefficient) across scored metrics of image quality, motion severity, and clinical interpretation, across each motion compensation technique.

Metric	No gating	Hard gating	Soft gating	XD‐GRASP	iMoCo
Diagnostic image quality	0.72	0.76	0.83	0.77	0.83
Extent of structural abnormality	0.90	0.70	0.85	0.83	0.80
Confidence in scoring	0.55	0.68	0.78	0.63	0.59
Cardiac motion severity	0.64	0.82	0.51	0.52	0.78
Respiratory motion severity	0.19	0.74	0.65	0.68	0.76
Clinical classification	0.83	0.98	0.91	0.91	0.82

Abbreviations: iMoCo, iterative motion compensation technique; XD‐GRASP, extra dimensional golden‐angle radial sparse parallel technique.

**FIGURE 4:**
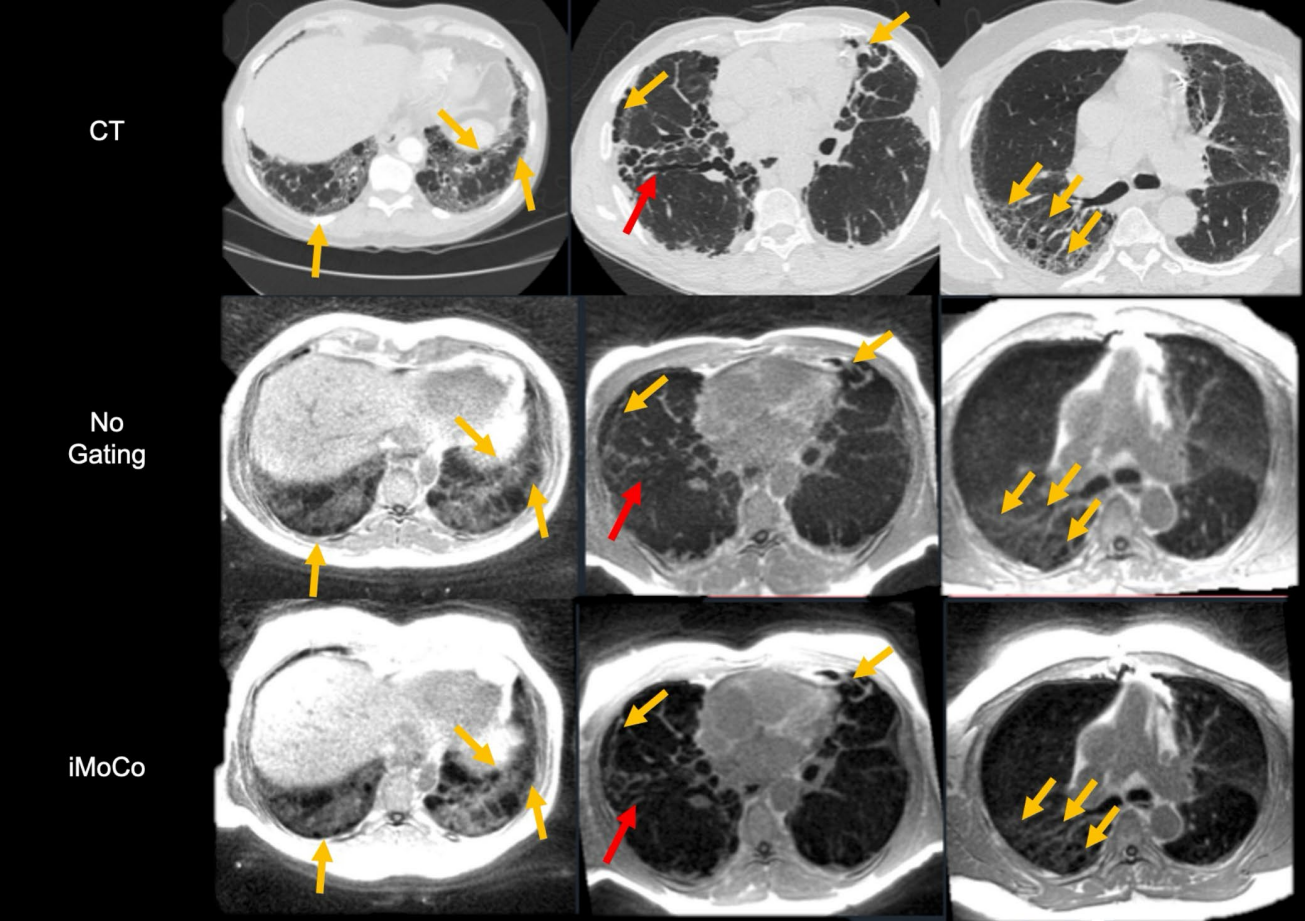
Improved visualization of clinically relevant parenchymal texture using iMoCo in patients with a diagnosis of IPF. Images of the lung parenchyma from three patients with patterns of “possible UIP,” “possible UIP,” and “definite UIP” are shown in columns A, B, and C respectively. Anatomically matched CT slices are shown in row 1, with corresponding UTE slices from no‐gating and iMoCo reconstructions in rows 2 and 3 respectively. Yellow arrows indicate locations of severe fibrotic changes distributed across the lung periphery, while the red arrow indicate airway structure, potentially suggesting traction bronchiectasis, visible on CT and iMoCo UTE, but obscured without motion compensation.

**FIGURE 5:**
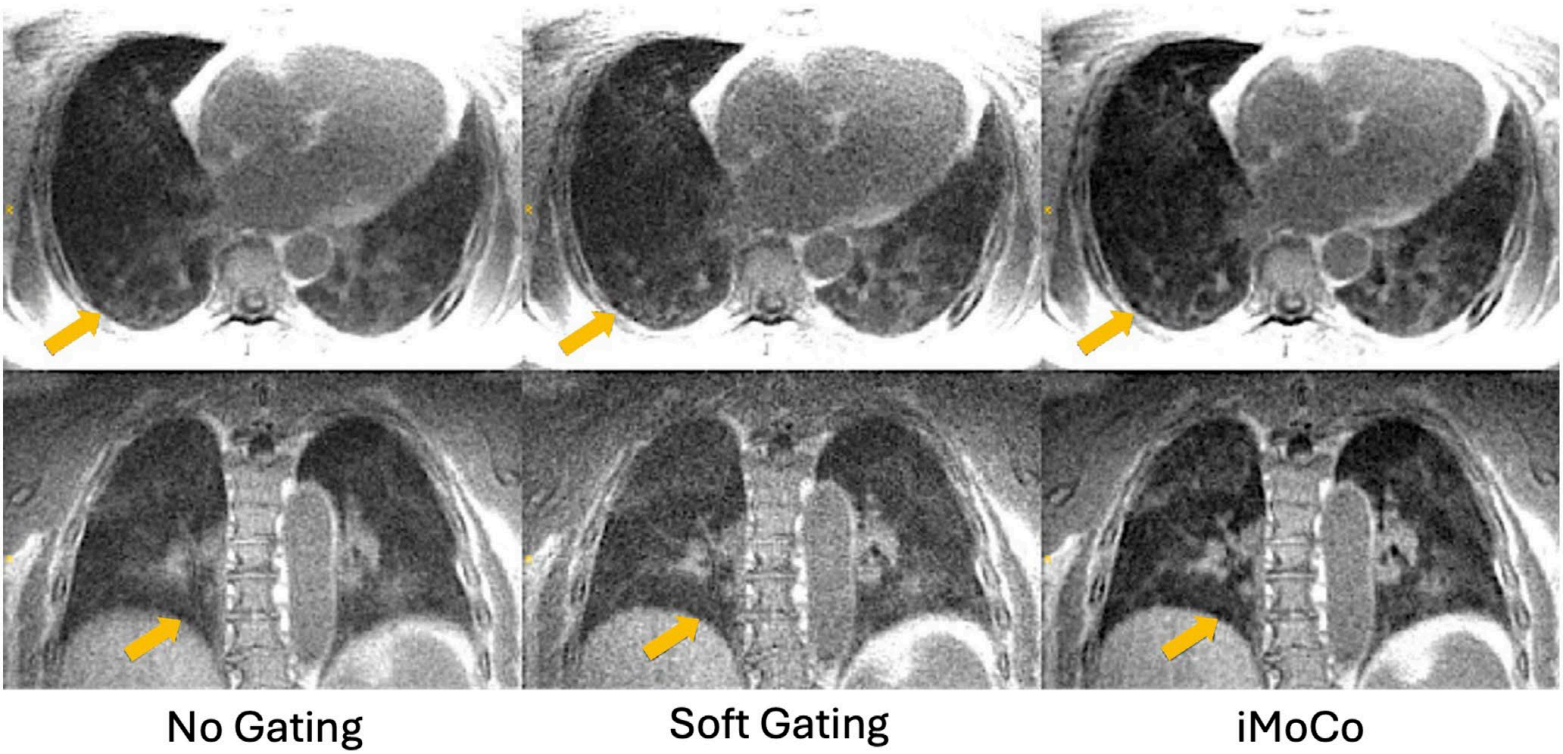
Sample datasets with differing clinical interpretation between readers. Images in each column show representative axial (top) and coronal (bottom) slices from three UTE motion mitigation schemes, acquired in a patient classified as possible UIP pattern from CT imaging. Two readers scored the “no gating” dataset as “unknown,” while the third reader scored it as “possible IPF.” With the soft‐gating reconstruction, the scores were reversed, with the first two readers scoring it as “possible IPF,” while the third reader scored it as “unknown.” With the improved contrast and visualization of regional patterns (examples shown in yellow arrows) from iMoCo, all readers agreed on a clinical classification of “possible IPF,” matching the CT designation.

Across the reconstruction schemes, iMoCo tended to perform the best and was ranked highest for image quality metrics in 55.5% of the cases. Qualitatively, compressed sensing (CS) approaches (XD‐GRASP and iMoCo) ranked highest across all reader scores of image quality in 74.6% of all datasets and in confidence of interpretation in 77.8% of all datasets. On average, both CS schemes also demonstrated better performance (lower scores) for both cardiac and respiratory motion severity, with iMoCo tending to outperform XD‐GRASP, although median scores were not significantly different from the gridding‐based schemes. Field strength did not emerge as a statistically significant variable for all quantitative and qualitative scores (lowest *p* = 0.37). In total, iMoCo showed overall the highest combination of CNR and sharpness with important strengths for indicating clinical patterns of fibrosis for diagnosis of IPF. Figure 4 depicts matched anatomic slices from corresponding HRCT images (top row). The severe fibrotic changes distributed across the periphery in the HRCT images are clearly visible in the iMoCo reconstructions but remain relatively obscured with the uncorrected images. Figure 5 demonstrates a useful example of blinded reader (dis)agreement on the clinical interpretation based on the UTE reconstructions while noting agreement on image quality. The CT pattern for this patient was scored as “possible UIP.” When reviewing the reconstruction without any motion mitigation, the classic features of basal predominant fibrosis were obscured to the extent that two readers scored this image as “unknown,” while the final reader scored it as “probable IPF.” In contrast, the soft‐gating approach resulted in the first two readers classifying the image as “probable IPF,” while the third reader regressed the score to “unknown.” These differences in interpretation were in spite of good agreement between readers on the image quality (2 for no‐gating, 2 for soft‐gating, and 3 for iMoCo in this case).

## 4 Discussion

The data from this study suggest that compressed sensing‐based motion compensation techniques provide significant improvement in metrics of image quality and in the clinical interpretation of pulmonary UTE‐MRI when evaluating structural pathology. Both XD‐GRASP and iMoCo techniques improve visualization of texture in the lung parenchyma and clinically relevant features typically visualized using HRCT.

The qualitative data suggest that after compensating for motion using conventional motion mitigation techniques, subtle but important features of texture in the lung parenchyma are lost when compared to the structural reference from corresponding CT images. Stable features such as extensive fibrotic burden, especially in the posterior lung, are likely less sensitive to motion‐related blurring and can be visualized with minimal motion compensation. However more subtle indications, such as honeycombing and reticulation which rely on the visualization of sharp air‐tissue boundaries, are lost with the gridding‐based techniques, impacted by low CNR, and significant undersampling as seen with hard‐gating. These pathological abnormalities are more likely to emerge in the CS‐based techniques, which provide the necessary contrast, preserve the edge information, without significant loss in SNR. This improvement in the image quality is likely due to a combination of improved data consistency through utilization of the spatio‐temporal correlations across the respiratory phases, image registration, and the efficient use of the full free‐breathing acquisition of *k*‐space data.

The qualitative visual evidence is supported by the significant improvement in quantitative metrics of CNR and sharpness. While XD‐GRASP and iMoCo images are comparable in metrics of sharpness, the CNR is significantly improved in iMoCo, suggesting that iMoCo reconstructions may be able to resolve clinically relevant features in parenchyma texture to a greater extent than the other techniques described in this study. A closer inspection of the impact of motion compensation is shown in Figure 4, demonstrating the differences between no gating and the iMoCo reconstructions. The parenchymal features near the cardiac and diaphragmatic boundaries are preserved with motion correction, allowing for better characterization of the local texture patterns. A small airway, which may be indicative of traction bronchiectasis (indicated with a red arrow), visible on the HRCT can be visualized in the iMoCo reconstruction but is largely obscured due to poor image quality without proper respiratory motion compensation. This feature is particularly interesting considering that the UTE‐MRI images are reconstructed at the end‐expiratory phase, while HRCT is acquired at full‐inspiration with patent airways. The improved tissue contrast with iMoCo results in sharper airway boundaries, allowing for easier evaluation of the airway structure in the context of the neighboring parenchyma, and the potential identification and diagnosis of clinically relevant features such as traction bronchiectasis and honeycombing. All the readers in this study indicated moderate‐to‐high scores in their confidence with the diagnostic classification using the CS‐based reconstructions, suggesting that the change in inflation state from the standard HRCT was not a limiting factor in their interpretation. The true impact of these motion mitigation approaches is demonstrated more clearly through the difference in interpretations shown in the example figures in Figure 5. The iMoCo reconstruction seems to have standardized the clinical interpretation, with all readers now agreeing on the “probable IPF” score. While visual scores of image quality may be similar, there is greater impact on the clinical interpretation, suggesting that the local SNR/CNR improvements support visual reads to a greater extent than suggested by scores of visual quality.

Expansion of free‐breathing UTE to clinical studies has several high‐impact applications. UTE datasets can be used to supplement HRCT in longitudinal studies, at shorter intervals than would be possible due to serial radiation exposures, increasing the utility of imaging‐based biomarkers in clinical trials evaluating acute progression and/or response to therapy. Using the HRCT as a structural reference and novel intensity normalization schemes can also help improve the regional quantitative assessment of parenchymal texture patterns similar to CT analysis pipelines, with potential for automated workflows and computer vision‐based algorithmic development. The free‐breathing approach also enables imaging with supplemental oxygen for patients, eliminates the need for exhaustive breath‐holding, and importantly, can provide dynamic data regarding the respiratory cycle. The CS‐based approaches are currently used to reconstruct at end‐expiration, but the other bins can be exploited to generate dynamic images which can reveal greater functional information than from a single virtual breath‐hold state. This dynamic information can also potentially help discriminate between localized disease patterns by visualizing structural and functional patterns such as air‐trapping, reticulation, and honeycombing.

## 5 Limitations

This study was based on a relatively small sample of 21 IPF patients of advanced age, utilizing images acquired at a single time point. While the sample size limits generalization to large cohort applications and immediate translation to clinical practice, the data provide a statistically significant rationale for further clinical evaluation of the free‐breathing UTE MRI technique in patient populations. Furthermore, while field strength did not emerge as a statistically significant variable, further work is needed to fully evaluate the impact of pulse sequence implementation across clinical systems from different vendors, utilizing different *k*‐space trajectories and scan parameters.

It should also be noted that the intrinsic spatial resolution of the UTE‐MRI images is substantially lower than HRCT, and because of the free‐breathing exam, the UTE MR images are presented at end‐expiration, as opposed to full‐inspiration in the case of HRCT. Specifically, the UTE MRI images were reconstructed to a voxel volume ~4× that in the HRCT images, and the true spatial resolution is likely closer to low‐dose CT protocols used for lung cancer screening and serial imaging needs. However, the promising results from this study suggest that despite these differences in spatial resolution and lung inflation, UTE‐MRI reconstructed with the XD‐GRASP/iMoCo approaches can serve as a tool for clinical diagnosis and longitudinal evaluation of relevant patterns of chronic fibrotic disease of the lung parenchyma. Both CS‐based techniques presented in this study are robust under steady cyclic respiratory motion, and will likely require further refinement to adjust for sudden/large changes in tidal volume, and/or bulk motion that may be likely in patients with severe disease. Furthermore, the compressed sensing‐based techniques require offline reconstruction with high performance computing resources, which can present a challenge in time‐sensitive clinical applications. However, this is unlikely to be the primary limiting factor in standard‐of‐care settings for chronic lung disease as increased computational resources become more routinely available clinically and reconstruction pipelines are improved using advanced techniques such as coil compression and coil sketching [40, 41].

## 6 Conclusion

Compressed sensing‐based methods for motion compensation provide significant improvement in metrics of image quality in pulmonary UTE‐MRI over conventional motion mitigation techniques. Review by expert radiologists demonstrates that the improvements in quantitative metrics of sharpness and contrast directly translate to improved clinical utility and increased confidence in diagnosing typical patterns of interstitial lung disease and IPF. Use of post‐processing steps to normalize intensity distributions between participants is likely to further improve regional characterization of the lung parenchyma and enable automated texture analysis workflows analogous to HRCT and expand the clinical relevance of UTE‐MRI in pulmonary applications.

## Funding

This work was supported by the National Institutes of Health, UL1TR000427, UM1TR004403; National Heart, Lung, and Blood Institute, HL126771, HL136965, HL169765, HL126774; NIH/NCATS, R01HL169765, S10OD026960, S10OD025025.

## Conflicts of Interest

S.B.F. serves as a scientific advisor for Polarean LLC and receives research funding from Polarean LLC, Siemens Healthineers INC, GE Healthcare INC, and Sanofi/Regeneron INC for development of pulmonary CT and MRI methods.
